# Factors Influencing Medical Students' Interest in Dermatology: A Cross-Sectional Study in the Eastern Province of Saudi Arabia

**DOI:** 10.7759/cureus.42313

**Published:** 2023-07-22

**Authors:** Muhannad M Alwadany, Heba Y Al Ojail, Abdulelah S Almousa, Feras M Al Wadany

**Affiliations:** 1 General Practice, King Faisal University, Hofuf, SAU; 2 Dermatology, King Faisal University, Hofuf, SAU

**Keywords:** cross-sectional study, saudi arabia, career choice, medical students, dermatology

## Abstract

Introduction

The demand for dermatologists is increasing due to the rising prevalence of skin diseases and the growing importance of dermatological care. However, there is limited research investigating the factors that influence medical students' interest in pursuing dermatology as a career option in Saudi Arabia, specifically in the Eastern Province.

Methods

This cross-sectional study aimed to assess the impact of dermatology rotation experience on the interest and perception of medical students and interns in the Eastern Province of Saudi Arabia. The participants consisted of medical students and interns located specifically in the Eastern Province. Data were collected through an online self-administered questionnaire that captured socio-demographic characteristics and evaluated the impact of dermatology rotation experience using a 3-point Likert scale. Convenient non-probability sampling was employed by sharing the questionnaire link on popular social media platforms.

Results

A total of 697 medical students from the Eastern Province of Saudi Arabia participated in this study, with an almost equal distribution between genders. A substantial proportion of participants expressed a strong preference for dermatology as their future career. While approximately 60% had completed a dermatology rotation, more than half found the process tiring. However, most participants agreed that dermatology offers flexible working hours, a better lifestyle, superior career options, and higher earnings compared to other healthcare professions. The study also revealed that factors such as age, marital status, academic level, GPA (grade point average), and income influenced the impact of the dermatology rotation.

Conclusion

This study sheds light on the factors influencing medical students' interest in dermatology and their perceptions of dermatology rotations. The findings emphasize the importance of diversity, early exposure, educational interventions, and supportive environments in promoting dermatology as a career choice. Overcoming barriers, enhancing transparency in assessment systems, and improving educational experiences can further foster interest in dermatology among medical students and address the growing demand for dermatological care.

## Introduction

In Saudi Arabia, as in many other countries, the demand for dermatologists has been increasing due to the rising prevalence of skin diseases and the growing importance placed on dermatological care [1]. Despite this demand, there is limited research exploring the factors that influence medical students' interest in dermatology as a career option in the Saudi Arabian context, particularly in the Eastern Province [1].

The choice of a medical specialty is a critical decision that medical students face during their education. The factors influencing students' career choices have been widely explored in the literature. In the context of dermatology, previous studies have identified several factors that shape medical students' interest in pursuing a career in dermatology [1,2].

One factor influencing career decisions is early exposure to dermatology during medical education. Research has shown that early exposure can positively impact students' perceptions and interest in a specific field [3-5]. Exposure to dermatology rotations provides students with the opportunity to gain firsthand experience in the field and develop a deeper understanding of the specialty. It allows them to interact with patients, witness the diversity of dermatological conditions, and observe the impact of dermatologists on patients' lives [3,6].

Furthermore, the portrayal of different medical specialties in the media can influence students' perceptions and career choices. The media often highlights certain specialties, potentially shaping students' perceptions of the attractiveness and prestige associated with those fields [1,7]. Additionally, factors such as lifestyle, career options, research opportunities, and financial considerations play a role in students' decision-making process. Medical students often consider the work-life balance, career prospects, and potential income when choosing a specialty [1]. The availability of supportive resources and mentorship can significantly impact students' career choices. The lack of support, equitable resources, and mentorship has been identified as a barrier faced by underrepresented minorities in pursuing dermatology residency programs [2].

Given the significance of dermatology in healthcare and the need for a diverse and well-prepared workforce, this study aims to explore the factors that influence medical students' interest in dermatology. By identifying these factors, medical educators, policymakers, and healthcare institutions can develop interventions and strategies to attract and retain medical students in dermatology.

## Materials and methods

Study design

This cross-sectional study aimed to evaluate the impact of dermatology rotation experience on the interest and perception of medical students and interns in the Eastern Province of Saudi Arabia.

Participants

The study population consisted of medical students and interns located in the Eastern Province of Saudi Arabia. Individuals from other regions, non-medical students, and those who refused to participate were excluded. The inclusion criteria encompassed medical students and interns specifically located in the Eastern Province.

Data collection

An online self-administered questionnaire was created using Google Forms to collect data. The questionnaire aimed to explore the impact of dermatology rotation experience on the interest and perception of medical students and interns in the Eastern Province. It was adapted from previous similar studies [8,9]. It included socio-demographic characteristics such as age group, sex, nationality, and residence. Additionally, questions related to the impact of dermatology rotation experience were included. Responses were measured using a 3-point Likert scale. Some questions were reverse-scored. The total impact score ranged from 1 to 60, with higher scores indicating a more positive impact on the dermatology rotation experience.

Sampling technique

Convenient non-probability sampling was employed to collect data. The online questionnaire link was randomly shared on various social media platforms, including Facebook, WhatsApp, Telegram, and Twitter. The platforms were chosen based on their popularity among medical students and interns in the Eastern Province. However, it is important to note that this sampling technique may introduce some selection bias and limit the generalizability of the findings.

Data analysis

The collected data were coded, entered, and analyzed using the Statistical Package for Social Science (SPSS) Version 23 (IBM Corp., Armonk, NY). Descriptive statistics, such as frequencies and percentages, were used to present quantitative data. Independent samples t-tests and one-way ANOVA (analysis of variance) tests were employed to examine quantitative data and compare differences in the impact of dermatology rotation experience among different groups.

Ethical considerations

Ethical approval for the study was obtained from the Research Ethics Committee at King Faisal University (approval number: KFU-REC-2023-JAN-ETHICS518). Informed consent was obtained from all participants before they could access the online questionnaire. Participants were provided with a clear explanation of the study objectives and assured that their participation was voluntary and anonymous. Strict confidentiality measures were implemented to protect participants' data, ensuring it was solely used for research purposes.

## Results

Characteristics of the study participants

A total of 697 medical students from the Eastern Province of Saudi Arabia participated in this study. The participants were evenly distributed between genders, with 52.2% being female and 47.8% being male. The majority of participants (44.2%) belonged to the age range of 21-23 years, while approximately one-third were in the age group of 18-20 years, and only 22.1% were between 24 and 26 years old. All study participants were Saudi Arabian, and the majority were single (89.8%). In terms of academic level, the highest percentage of participants were in their fifth year (22.5%), and approximately 20.1% were in the internship phase. Furthermore, 56.2% of participants had a GPA (grade point average) score of more than 4.5 out of 5.0. Regarding average monthly income, 73.5% of participants reported earning between 1,000 and 3,999 SAR (Table 1).

**Table 1:** Socio-demographic characteristics of the study participants GPA, grade point average; SAR, Saudi Riyal

Variable	Categories	Frequency	Percent
Gender	Male	333	47.8
	Female	364	52.2
Age (years)	18-20	235	33.7
	21-23	308	44.2
	24-26	154	22.1
Nationality	Saudi	697	100
	Non-Saudi	0	0
Marital status	Single	626	89.8
	Married	71	10.2
Year of medical educational	First year	69	9.9
	Second year	66	9.5
	Third year	100	14.3
	Fourth year	112	16.1
	Fifth year	157	22.5
	Sixth year	53	7.6
	Intern	140	20.1
GPA	Less than 4.0	28	4
	4.0–4.5	277	39.7
	More than 4.5	392	56.2
Average monthly income (SAR)	Less than 1,000	132	18.9
	1,000–3,999	512	73.5
	4,000 or more	53	7.6

Dermatology rotation impact

The results provided significant insights into the impact of the dermatology rotation on medical students' preferences and perceptions. A substantial proportion of study participants (70.9%) expressed a strong preference for dermatology as a future career.

While nearly 60% of participants had enrolled in a dermatology rotation, more than half of them found the enrollment process or studying for a dermatology rotation to be tiring (59.8%). However, the majority (69.2%) agreed that working hours in dermatology are flexible.

Most participants (70.9%) believed that dermatologists enjoy a better lifestyle compared to other healthcare professionals and have superior career options. Similarly, the same percentage thought that dermatologists earn more than other healthcare professionals.

A significant proportion of participants (62.1%) believed that the length of dermatology residency may influence medical students' career choices and that dermatologists have a greater impact on patients' lives. Additionally, more than half of the participants believed that research opportunities are more prevalent in the field of dermatology.

Around two-thirds of respondents agreed that dermatology rotations provide exposure to a more diverse group of patients. The majority of participants (70.9%) agreed that dermatology rotations offer greater exposure to a wide variety of medical issues and more opportunities for long-term patient relationships.

A significant proportion (69%) of participants found the enrollment process in a dermatology residency program to be difficult. Only 13.8% perceived dermatology rotations as more stressful, while 27.1% disagreed with this statement. Conversely, 65.3% of respondents believed that there are fewer chances of medical errors in the field of dermatology.

Most participants (70.9%) expressed a desire to pursue dermatology as a career, and the majority believed that early training in dermatology during medical education would help students make better decisions about their future specialty. Furthermore, the majority of study participants (70.9%) expressed interest in attending a dermatology pre-clerkship session if offered at their institution (Table 2).

**Table 2:** Influence of dermatology rotation experience on medical students' interest and perception

Statement	Agree	Disagree	Not sure
I prefer dermatology as my future career	494 (70.9)	97 (13.9)	106 (15.2)
Dermatology offers more opportunities in the field of research	382 (54.8)	79 (11.3)	236 (33.9)
I have enrolled in or studied for a dermatology rotation	417 (59.8)	66 (9.5)	214 (30.7)
Dermatologists enjoy a better lifestyle compared to other healthcare professionals	494 (70.9)	79 (11.3)	124 (17.8)
Dermatology rotations provide exposure to more diverse groups of patients	463 (66.4)	79 (11.3)	155 (22.2)
I find dermatology rotations tiring	408 (58.5)	123 (17.6)	166 (23.8)
After completing a dermatology rotation, I feel that I want to pursue dermatology as my future career	494 (70.9)	91 (13.1)	112 (16.1)
Working hours in dermatology are flexible	482 (69.2)	79 (11.3)	136 (19.5)
There are fewer chances of medical errors in the field of dermatology	455 (65.3)	51 (7.3)	191 (27.4)
The length of a dermatology residency may affect medical students' choice for their future career	433 (62.1)	79 (11.3)	185 (26.5)
An early training session about dermatology during medical education will help students make a better decision about their future specialty	494 (70.9)	91 (13.1)	112 (16.1)
I think dermatologists have a greater impact on patients' lives	429 (61.5)	79 (11.3)	189 (27.1)
Dermatology rotations provide greater exposure to a wide variety of medical issues	494 (70.9)	79 (11.3)	124 (17.8)
I would attend a dermatology pre-clerkship session if offered at my institution	494 (70.9)	63 (9)	140 (20.1)
Dermatology rotations provide more opportunities for long-term patient relationships	494 (70.9)	79 (11.3)	124 (17.8)
It is difficult to get enrolled in a dermatology residency program	481 (69)	79 (11.3)	137 (19.7)
Dermatology rotations are more stressful	96 (13.8)	189 (27.1)	412 (59.1)
I feel that dermatologists have better career options	494 (70.9)	79 (11.3)	124 (17.8)
Dermatologists earn better than other healthcare professionals	494 (70.9)	79 (11.3)	124 (17.8)

Relationship between level of impact and socio-demographic factors

Table 3 summarizes the relationship between the level of impact of the dermatology rotation and socio-demographic factors. The average score for the impact of the dermatology rotation on the interest of medical students was 48.8 ± 9.8 (range: 27-56) out of a total score of 60. The results indicated a significant difference in the impact of the dermatology rotation on the interest of female students compared to male students (mean scores = 50.1 vs. 47.4, respectively) (P < 0.001). The perception of students aged between 24 and26 years, married participants, students in their sixth year and internship phase, students with a GPA of less than 4.0, and those with a monthly income of 10,000 SAR or more were significantly affected by the dermatology rotation (P < 0.001).

**Table 3:** Relationship between the level of Impact and various socio-demographic factors GPA, grade point average; SAR, Saudi Riyal

Variable	Categories	Impact score		P-value
		Mean	SD	
Gender	Male	47.4	11.53	<0.001
	Female	50.1	7.73	
Age (years)	18-20	46.6	10.47	<0.001
	21-23	47.4	10.56	
	24-26	54.7	0.60	
Marital status	Single	48.1	10.16	<0.001
	Married	54.4	0.78	
Year of medical educational	First year	45.0	8.85	<0.001
	Second year	42.9	13.71	
	Third year	50.2	7.59	
	Fourth year	49.5	6.33	
	Fifth year	46.2	13.18	
	Sixth year	54.5	0.87	
	Intern	52.5	5.30	
GPA	Less than 4.0	53.6	5.58	<0.001
	4.0–4.5	45.0	0.51	
	More than 4.5	45.0	5.58	
Average monthly income (SAR)	Less than 1,000	51.0	6.68	<0.001
	1,000–3,999	47.6	10.66	
	10,000 or more	54.5	0.87	

## Discussion

The findings of this study provide valuable insights into the factors influencing medical students' interest in dermatology as a career choice and their perceptions of dermatology rotations. Several previous studies have explored related aspects, which further support and contextualize our findings.

Diversity within the field of dermatology has been a topic of concern, with dermatology being considered one of the least diverse fields of medicine [10]. Vasquez et al. [2] identified various barriers faced by underrepresented minority applicants in dermatology residency programs in the United States, including lack of support, equitable resources, mentorship, and financial constraints. These barriers highlight the importance of addressing systemic factors to promote diversity and inclusivity in dermatology [11,12].

The experiences of medical students during dermatology clerkships have also been investigated. Yoon et al. [13] found that students had a limited understanding of the assessment systems prior to the clerkship and perceived them as unclear, non-transparent, and subjective. This aligns with our findings, where students found the enrollment process and studying in dermatology residency programs to be difficult. Improving transparency and clarity in the assessment and enrollment processes can enhance students' experiences and facilitate their engagement in dermatology.

Efforts to improve dermatology education and students' perceptions have been explored in various studies. Blakely et al. [1] demonstrated a positive impact of a dermatology pre-clerkship intervention on students' comprehension of the subject. These findings emphasize the importance of early exposure and comprehensive educational interventions to enhance students' understanding and interest in dermatology.

The influence of clinical rotations on career choices has been studied in different specialties. Marshall et al. [14] conducted a systematic review and found that surgical rotations had a positive impact on students' consideration of surgery as a future career. Similarly, our study revealed that dermatology rotations significantly influenced medical students' interest in pursuing dermatology as a career. These findings highlight the value of clinical experiences in shaping students' career decisions and the need for targeted interventions in specialty-specific rotations.

The factors influencing medical students' choice of dermatology as a specialty have been explored in various contexts. Some studies identified the difficulty of gaining entry into a dermatology residency program as a major barrier, while factors such as the prestige of being a dermatologist, lifestyle satisfaction, clinical skills utility, and research opportunities were attractive aspects. Our findings corroborate these factors, indicating that students in Saudi Arabia also consider similar elements when contemplating dermatology as a future career [8,9].

The effectiveness of professional development programs in shaping medical education has also been investigated. Alwazzan et al. [15] highlighted the efficacy of an identity formation program in dermatology residency, emphasizing the transformational changes that can occur at both individual and professional levels. This suggests that targeted interventions and innovative educational approaches can play a crucial role in shaping students' perceptions and career choices.

Limitations of the study should also be acknowledged. Firstly, the study used a convenient non-probability sampling technique, which may introduce selection bias and limit the generalizability of the findings to the broader population of medical students and interns in the Eastern Province of Saudi Arabia. Additionally, the reliance on self-reported data through an online questionnaire may be subject to response biases and inaccuracies. The cross-sectional design of the study limits our ability to establish causality and determine long-term effects. Furthermore, the study focused on the Eastern Province of Saudi Arabia, and the findings may not be applicable to other regions or countries with different healthcare systems and cultural contexts. Future research could employ a more diverse and representative sample, employ longitudinal designs, and consider multi-center studies to enhance the generalizability and depth of understanding of the factors influencing medical students' interest in dermatology.

## Conclusions

In conclusion, our study contributes to the existing literature by providing insights into the factors influencing medical students' interest in dermatology and their perceptions of dermatology rotations. The findings align with previous research that emphasizes the importance of diversity, early exposure, educational interventions, and supportive environments in promoting dermatology as a viable career option. Addressing barriers, improving transparency in assessment systems, and enhancing educational experiences can foster greater interest in dermatology among medical students and help meet the growing demand for dermatological care.
